# Non-Lethal Effects of *N*-Acetylcysteine on *Xylella fastidiosa* Strain De Donno Biofilm Formation and Detachment

**DOI:** 10.3390/microorganisms7120656

**Published:** 2019-12-05

**Authors:** Cristina Cattò, Luca De Vincenti, Francesca Cappitelli, Giusy D’Attoma, Maria Saponari, Federica Villa, Fabio Forlani

**Affiliations:** 1 Department of Food, Environmental and Nutritional Sciences, Università degli Studi di Milano, via Celoria 2, 20133 Milano, Italy; cristina.catto@unimi.it (C.C.); luca.devincenti@unimi.it (L.D.V.); francesca.cappitelli@unimi.it (F.C.); fabio.forlani@unimi.it (F.F.); 2 Institute for Sustainable Plant Protection, Consiglio Nazionale delle Ricerche, via Amendola 165/A, 70126 Bari, Italymaria.saponari@ipsp.cnr.it (M.S.)

**Keywords:** *N*-acetylcysteine, non-lethal concentration, biofilm formation, *Xylella fastidiosa* strain De Donno, detachment

## Abstract

This study investigated in-vitro the non-lethal effects of *N*-acetylcysteine (NAC) on *Xylella fastidiosa* subspecies *pauca* strain De Donno (Xf-DD) biofilm. This strain was isolated from the olive trees affected by the olive quick decline syndrome in southern Italy. Xf-DD was first exposed to non-lethal concentrations of NAC from 0.05 to 1000 µM. Cell surface adhesion was dramatically reduced at 500 µM NAC (−47%), hence, this concentration was selected for investigating the effects of pre-, post- and co-treatments on biofilm physiology and structural development, oxidative homeostasis, and biofilm detachment. Even though 500 µM NAC reduced bacterial attachment to surfaces, compared to the control samples, it promoted Xf-DD biofilm formation by increasing: (i) biofilm biomass by up to 78% in the co-treatment, (ii) matrix polysaccharides production by up to 72% in the pre-treatment, and (iii) reactive oxygen species levels by 3.5-fold in the co-treatment. Xf-DD biofilm detachment without and with NAC was also investigated. The NAC treatment did not increase biofilm detachment, compared to the control samples. All these findings suggested that, at 500 µM, NAC diversified the phenotypes in Xf-DD biofilm, promoting biofilm formation (hyper-biofilm-forming phenotype) and discouraging biofilm detachment (hyper-attachment phenotype), while increasing oxidative stress level in the biofilm.

## 1. Introduction

*Xylella fastidiosa* is a bacterium that infects more than 500 different plants including ornamental plants, landscape trees, wild and crop species [1,2]. Serious economic losses caused by *X.-fastidiosa*-related epidemic diseases in valuable crops have been well documented [3]. The first field outbreak of *X. fastidiosa* in the European Union dated back in October 2013, when the Italian Phytosanitary Authorities reported the presence of this quarantined pathogen in southern Italy’s olive orchards [4]. The novel *X. fastidiosa*-related disease called olive quick decline syndrome (OQDS), known to cause severe leaf scorth and branch dieback, poses one of the most debilitating threats to the European agriculture and landscape [5,6]. *X. fastidiosa* subspecies *pauca* strain De Donno (Xf-DD) was recovered in 2014 from OQDS-affected olive trees. Its complete genome has been sequenced [7] and the pathogenicity on the olive trees and other susceptible hosts was confirmed by needle inoculations [8].

The major pathogenic mechanism of *X. fastidiosa* relies on the obstruction of xylem vessels caused by biofilm formation [9,10]. A biofilm is a complex community of microorganisms that are attached to surfaces (in this case, on the cuticular lining of the insect foregut and/or the wall of the xylem), embedded in a self-produced matrix of hydrated extracellular polymeric substances, EPS [11]. Within the biofilm, the cells coordinate their behavior and function to survive and to persist in their environment. Biofilm formation in the insect foregut is closely associated with the acquisition, retention and transmission of the phytopathogen by sharpshooter vectors [12]. While in plants, the biofilm clogs the xylem vessels, depriving the plants of water and nutrition, and ultimately leading them to their death [13]. 

So far, there has been neither a therapy to cure *Xylella*-associated diseases nor a strategy to contain its impact. The spread of the disease is minimized by simply removing the infected plants and reducing the vector populations [1]. However, intense research programs are ongoing worldwide to search for remedies for this bacterial infection, which include, but are not limited to, the search for natural enemies to control the vectors [14,15], the production of transgenic plants [16] and the induction of plant defense responses [17].

Other studies have reported some strategies that directly target the pathogen to reduce its spread inside the plant. These strategies are aimed to kill the phytopathogen by using specific phages that are able to lysate *X. fastidiosa* cells [18], antibiotics such as tetracyclines [19,20], metal compounds like copper-zinc fertilizers [21,22], and some natural products such as polyphenols, azadirachtin A, hesperidin and radicinin [23,24,25]. 

Among natural bioactive molecules, *N*-acetylcysteine (NAC), a thiol-containing compound commonly found in *Allium* plants, has shown interesting antibiofilm activities in clinical settings [26,27,28]. Muranaka et al. [29] and Picchi et al. [30] reported promising results in treating *X. fastidiosa* strain 9a5c, isolated from citrus variegated chlorosis (CVC) symptomatic sweet orange trees, and other phytopathogenic bacteria with lethal NAC concentrations. In-vitro studies have shown that concentrations of NAC of 1.0, 2.0 and 6.0 mg/mL affect *X. fastidiosa* strain 9a5c adhesion to glass surfaces and EPS amount, consequently reducing the biofilm biomass [29]. The reduction in biofilm formation was correlated to the reduction in the number of viable cells, suggesting that even the lowest NAC concentration tested (1 mg/mL) had a biocidal effect on *X. fastidiosa* strain 9a5c [29]. In-vivo experiments have revealed that orange plants treated with lethal concentrations of NAC supplied in hydroponics and fertigation systems, as well as adsorbed in organic fertilizer, display significant CVC symptom remission and bacterial population reduction [29]. 

Despite the encouraging results, the non-lethal effects of NAC on *X. fastidiosa* has never been investigated. This is an important aspect to be studied, because for each biocide treatment, the concentration of the biocide is reasonably expected to diminish over time and space, resulting in a non-lethal level away from the application point. The physiological and behavioral responses of *X. fastidiosa* biofilm at this low concentration may be different from those at biocidal concentrations. 

With these considerations, the main goal of this study was to investigate in-vitro the non-lethal effects of NAC on *X. fastidiosa* subspecies *pauca* strain De Donno (Xf-DD) biofilm, originally isolated from OQDS-affected olive trees in southern Italy. After the response of Xf-DD to a range of non-lethal concentrations was screened, 500 µM NAC showed the highest effect on reducing the number of cells attached to surfaces, and hence, this concentration was selected for further investigations. Additional biofilm experiments with 500 µM NAC simulated three different treatments: (i) pre-treatment, where planktonic cells were treated with NAC before their attachment to the surface; (ii) post-treatment, where biofilm was grown in the presence of NAC after the attachment phase; and (iii) co-treatment, where planktonic and biofilm cells were exposed to NAC. For the first time, the effects of 500 µM NAC on Xf-DD biofilm were studied, taking into consideration biofilm formation and detachment. To this end, biofilm physiology and structural development, modulation of oxidative homeostasis, and induction of biofilm dislodgment were investigated.

## 2. Materials and Methods 

### 2.1. Bacterial Strain and Culture Conditions 

Xf-DD strain (CFBP 8402) was originally isolated in 2014 from a OQDS-affected olive tree (Olea Europaea cv Ogliarola) grown in the municipality of Gallipoli (Apulia region, southern Italy) [31]. The strain was obtained from olive cuttings on buffered charcoal yeast extract (BCYE) agar medium [32], then stored at −80 °C in 50% glycerol and was routinely grown on PD3 medium for 8–10 days at 28 °C.

### 2.2. Chemical Compound

*N*-acetylcysteine (NAC) was purchased from Sigma-Aldrich (A7250, ≥99% purity grade). NAC was dissolved in deionized water at room temperature to a concentration of 5000 µM. After adjusting pH to 6.8 with sodium hydroxide (1 M NaOH), the stock solution was sterilized by filtration through a 0.22 µm filter and diluted in PD3 medium to the final concentrations of 1000, 500, 250, 50, 5, 0.5 and 0.05 µM. 

### 2.3. Toxicity Assay in the Presence of NAC

The ability of 1000 µM of NAC to inhibit the planktonic growth of Xf-DD was investigated as previously reported by Kandel et al. [33], with some modification. Briefly, Xf-DD colonies grown on PD3 agar plates for 8–10 days at 28 °C were scraped and resuspended in one mL of PD3 broth. Ten µL of cell suspension were diluted in PD3 (initial optical density of 0.04 at 600 nm) supplemented with 0 (negative control) and 1000 µM of NAC to obtain a final volume of 200 µL. The suspensions were placed in transparent 96-well polystyrene-based microtiter plates (Thermo-Fisher Scientific, Waltham, MA, USA). The plates were incubated at 28 °C, with shaking at 150 rpm/min. Growth curves were generated by measuring the optical density at 600 nm (OD_600_) every 24 h for 7 days using an Infinite F200 PRO microplate reader (TECAN, Mannedorf, Switzerland). The polynomial Gompertz model was used to fit the growth curves and the maximum specific growth (OD_600_/day) was calculated using the GraphPad Prism software (version 5.0, San Diego, CA, USA). Experiments were repeated independently three times with at least six technical replicates per time.

### 2.4. Xf-DD Adhesion in 96-Well Plates 

Xf-DD colonies grown on PD3 agar plates were scraped and resuspended in 2 mL of PD3 (optical density at 600 nm of 0.8). Sterile polystyrene 96-well plates containing 190 μL PD2 per well supplemented with 0, 1000, 500, 250, 50, 5, 0.5 and 0.05 µM NAC were inoculated with 10 μL of cell suspensions. After 1 day of incubation at 28 °C, the cells suspensions were removed, the wells rinsed 3 times with Milli-Q water and stained with 0.1% crystal violet to quantify the attached cells [34]. The stain was removed after 30 min, the adhered cells were gently washed 3 times with Milli-Q water and the microtiter plate dried for 2 h. Crystal violet was then solubilized with 200 μL of 6:4 acetone:ethanol. The absorbance at 550 nm was read using the Infinite F200 PRO microplate reader (Tecan, Mannedorf, Switzerland). All experiments were replicated at least three times.

### 2.5. Xf-DD Biofilm Growth at the Solid/Air Interface 

Xf-DD biofilm was grown on a transwell device as previously described by Garuglieri et al. [35] with some modifications. Briefly, Xf-DD colonies grown on PD3 agar plates were scraped and resuspended in 2 mL of PD3 broth without (–) and with 500 µM of NAC (NAC). After 24 h of incubation at 28 °C under agitation at 140 rpm, 50 µL of bacterial broth culture (0.5 optical density at 600 nm) were inoculated at the center of a sterile polycarbonate membrane (Whatman Nucleopore, diameter 2.5 cm, pore diameter 0.2 μm) and, once the inoculum was completely dried, the membrane was carefully transferred to a transwell device (ThinCertTM Cell Culture Inserts with translucent PET membrane—Greiner Bio-One, Kremsmünster, Austria) inlaid in a 6 well culture plate (Greiner Bio-One, Kremsmünster, Austria). One mL of PD3 medium without (–) and in the presence of 500 µM of NAC (NAC) was added to inoculate the plate well. Mediums were replaced every 24 h. Biofilms were grown at 28 °C for 72 h. 

**Figure 1:**
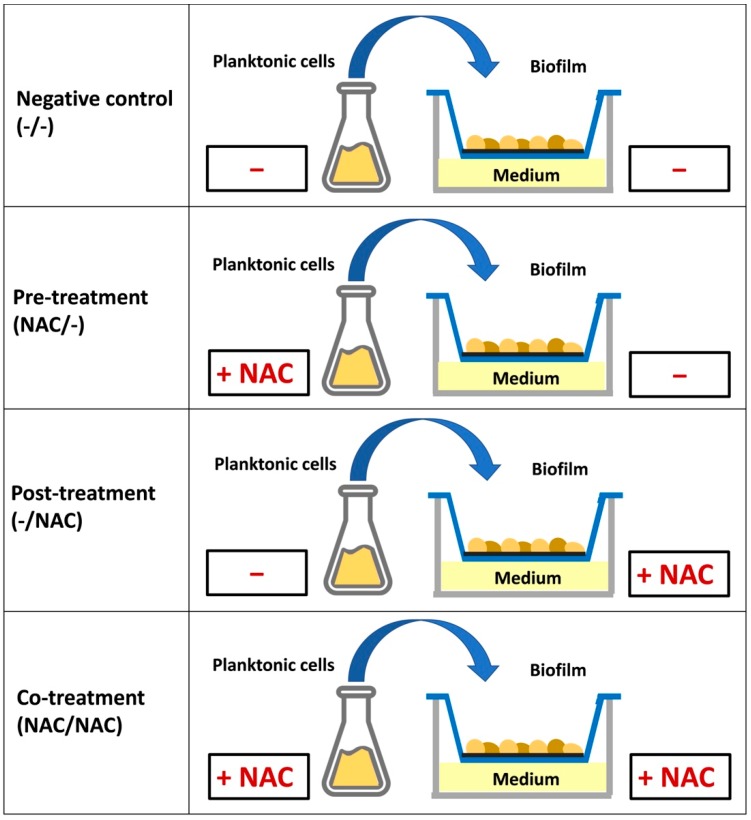
*N*-acetylcysteine (NAC) treatments used in this study. –/–: control, both planktonic and biofilm cells grown without NAC; NAC/–: pre-treatment, only the planktonic cells grown with NAC; –/NAC: post-treatment, only the biofilm cells grown with NAC; NAC/NAC: co-treatment, both planktonic and biofilm cells grown with NAC.

Xf-DD biofilm was grown in four different conditions (Figure 1): (1) both planktonic and biofilm cells grown without NAC (named –/–), negative control; (2) planktonic cells treated with 500 µM NAC and biofilm cells grown without NAC (named NAC/–), pre-treatment; (3) planktonic cells grown without NAC and biofilm cells grown with 500 µM NAC (named –/NAC), post-treatment; (4) both planktonic and biofilm cells grown with 500 µM of NAC (named NAC/NAC), co-treatment. After 7 days, biofilms obtained from each treatment were collected for further analyses. 

### 2.6. Biofilm Biomass Quantification 

Biofilm biomass was evaluated measuring the amount of cellular protein [36]. Briefly, two membranes for each treatment were transferred in 1 mL of phosphate buffered saline solution (PBS), and biofilm was removed from the membrane surface by 1 min vortex mixing, 2 min sonication (50% amplitude, in a water bath; Branson 3510, Branson Ultrasonic Corporation, Dunburry, CT, USA) followed by another 1 min of vortex mixing. Membranes were subsequently removed from the suspension and cells were broken by sonication (three cycles of 30 s at 40% power sonication with 15 s intervals; Branson 3510, Branson Ultrasonic Corporation, Dunburry, CT, USA) followed by centrifugation (15 min at 4 °C at 5700× *g*). The supernatant was collected and the protein amount was quantified by Bradford assay [37] using bovine serum albumin as standard. Obtained data were normalized against the membrane area, and the means reported. The experiments were repeated three times with at least three technical replicates.

### 2.7. Live/Dead Biofilm Assay 

Seven-day biofilms grown on the membrane in the transwell devices were transferred in 1 mL of Milli-Q water. Biofilms were dislodged from the membranes and cells were broken as reported in the section ‘Biofilm biomass quantification’. The Live/Dead BacLight viability kit (Molecular Probes—Life Technologies, Carlsbad, CA, USA) was used to detect live and dead cells according to the manufacturer’s instructions. The fluorescence intensity was measured using the Infinite 200 PRO Microplate Reader (Tecan, Manneford, Switzerland) with excitation at 480 nm and emission at 516 nm for the live green cells, and excitation at 581 nm and emission at 644 nm for the red dead cells. Fluorescence intensity was normalized by the proteins within the biofilm, divided for the area of the membrane, and the means reported. Relative viability within the biofilms was calculated by dividing the fluorescent intensity of normalized live cells by the fluorescent intensity of normalized dead cells in each sample. Experiments were repeated three times with at least six technical replicates per time. 

### 2.8. Confocal Laser Scanning Microscope (CLSM) 

Three-dimensional morphology of seven day-biofilms from each treatment were analyzed by CLSM. The biofilms were stained with Sybr green I fluorescent nucleic acid dye (S7563, Thermo Fisher Scientific, Waltham, MA, USA) to reveal biofilm cells. The staining was performed by incubating the biofilm with 200 µL of 1:1000 Sybr green I dye solution in PBS at room temperature in the dark for 30 min, and then rinsed with PBS. Membranes without biofilms were also stained in order to exclude any false positive signals. Each biofilm sample was visualized using a Nikon A1 with excitation at 488 nm line, and emission at 500 to 550 nm (green channel). Images were captured with a 100× oil immersion objective and analyzed with the software Fiji [38].

### 2.9. Extraction and Characterization of the Extracellular Polymeric Substances (EPS) Matrix 

Seven day-biofilms from each treatment were analyzed for their EPS proteins and polysaccharides content. EPS extraction was performed as reported by Villa et al. [39]. The Bradford and the phenol-sulfuric acid [37,40] methods were applied for quantification of proteins and polysaccharides using bovine serum albumin and glucose as standard, respectively. Obtained data were normalized by the area and the means reported. The EPS-polysaccharide/EPS-protein ratios were also calculated. The experiments were repeated three times with at least six technical replicates per time.

### 2.10. Oxidative Stress Assay 

Seven day-biofilms from each treatment were transferred in 1 mL of 50 mM PBS and the biofilm was detached from the membranes by vortex mixing and sonication as described above in the section ‘Biofilm Biomass Quantification’. The suspension was centrifuged for 20 min at 11,000× *g*. The supernatant was transferred to another tube, filtered (0.22 µm pores size) and analyzed for the extra-cellular ROS content. The pellet was resuspended in 50 mM PBS, cells were broken by sonication as described previously in the section ‘Biofilm Biomass Quantification’, and the intra-cellular ROS content was quantified. Intra- and extra-cellular oxidative stresses were quantified using the ROS sensitive probe 2,7-dichlorofluorescein-diacetate according to Jakubowski et al. [41]. Fluorescence was measured using a microplate reader (Tecan, Manneford, Switzerland) at excitation wavelength of 485 nm and emission wavelength of 535 nm. Obtained data were normalized by the protein within the biofilm, divided for the area, and the means reported. Experiments were repeated three times with at least five technical replicates per time.

### 2.11. Biofilm Dispersion

Membranes with biofilms grown for 7 days under the different treatments were transferred in petri dishes (ø 60 mm) and incubated with 2 mL of phosphate buffered saline solution (PBS, Sigma-Aldrich, pH 6.8 adjusted by potassium hydroxide) or 500 µM NAC for further 24 h at 28 °C. Subsequently, biofilms on the membranes and those dislodged in the bulk liquid were analyzed for their biomass and the level of ROS as previously described in ‘Biofilm Biomass Quantification’ and ‘Oxidative Stress Assay’. The detachment index was calculated as: (protein amount in the detached biofilm × 100)/(protein amount detached biofilm + protein amount in the biofilm on the membrane). Experiments were repeated independently three times with at least four technical replicates per time.

### 2.12. Statistical Analysis 

Two-tailed ANOVA and Student’s *t*-test analysis, via a software run in MATLAB environment (Version 7.0, The MathWorks Inc., Natick, MA, USA), were applied to statistically evaluate any statistically significant differences among the samples. ANOVA and Student’s *t*-test analysis were carried out after verifying data independence (Pearson’s chi-square test), normal distribution (D’Agostino-Pearson normality test) and homogeneity of variance (Bartlett’s test). Tukey’s honestly significant different test (HSD) was used for pairwise comparison to determine the significance of the data. Statistically significant results were depicted by *p*-values ≤ 0.05.

## 3. Results

### 3.1. 1000 µM NAC Did Not Affect Xf-DD Planktonic Growth

**Figure 2:**
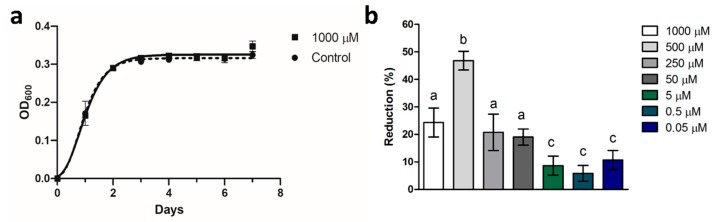
(**a**) OD_600_ values and Gompertz growth curves of *Xylella fastidiosa* subspecies *pauca* strain De Donno (Xf-DD) without and with 1000 µM NAC. Data represent the mean ± SD of three independent measurements. (**b**) Percentage of inhibition of Xf-DD attachment exposed to non-lethal concentrations of NAC. Data represent the mean ± SD of three independent measurements. Different superscript letters indicate statistically significant differences (Tukey’s honestly significant different test (HSD), *p* ≤ 0.05) between conditions.

Figure 2a shows the 7 day-growth curves of Xf-DD in the presence and absence of 1000 µM NAC. The results indicated no statistically significant differences on the maximum specific growth rate (OD_600_/day) between the NAC-treated samples (0.218 ± 0.01; R^2^ = 0.986) and control samples (0.234 ± 0.02; R^2^= 0.972). Therefore, 1000 µM NAC was considered a non-lethal concentration.

### 3.2. Non-Lethal Concentrations of NAC Affected Xf-DD Adhesion in 96-Well Plates 

Figure 2b displays the inhibition percentage of Xf-DD adhesion induced by different non-lethal concentrations of NAC compared to the control samples. Although all the non-lethal concentrations affected Xf-DD attachment, 500 µM NAC showed the highest effects of reducing cell adhesion by 47%. The non-lethal concentration of 500 µM NAC was used in the subsequent studies. 

### 3.3. NAC Treatments Increased Xf-DD Biofilm Biomass, While Not Affecting Cell Viability 

**Figure 3:**
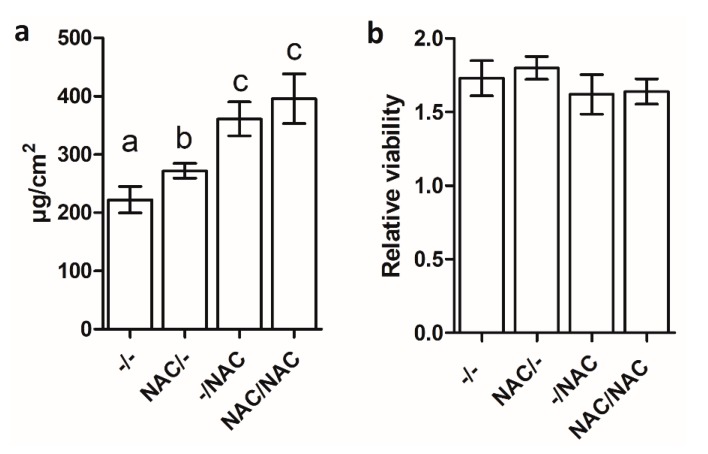
(**a**) Biofilm biomass expressed as protein amount of Xf-DD biofilm grown for 7 days under different conditions. (**b**) Relative viability of Xf-DD biofilm grown under different treatments. Data represent the mean ± SD of three independent replicates. Different superscript letters indicate statistically significant differences (Tukey’s HSD, *p* ≤ 0.05) between conditions. –/–: control, both planktonic and biofilm cells grown without NAC; NAC/–: pre-treatment, only the planktonic cells grown with NAC; –/NAC: post-treatment, only the biofilm cells grown with NAC; NAC/NAC: co-treatment, both planktonic and biofilm cells grown with NAC.

The effects of 500 µM NAC on Xf-DD biofilm biomass were assessed by quantifying the protein amount within the biofilm. The data showed that 500 µM NAC significantly increased the amount of biofilm proteins in all the treatments, compared to the control (Figure 3a), highlighting the maximum increase for the treatment NAC/NAC (78%) and –/NAC (62%), followed by NAC/– (22%).

The Live/Dead viability assay was performed to verify the ability of NAC to affect biofilm viability. Relative viability calculated for each condition revealed no statistically significant differences among samples (Figure 3b).

**Figure 4:**
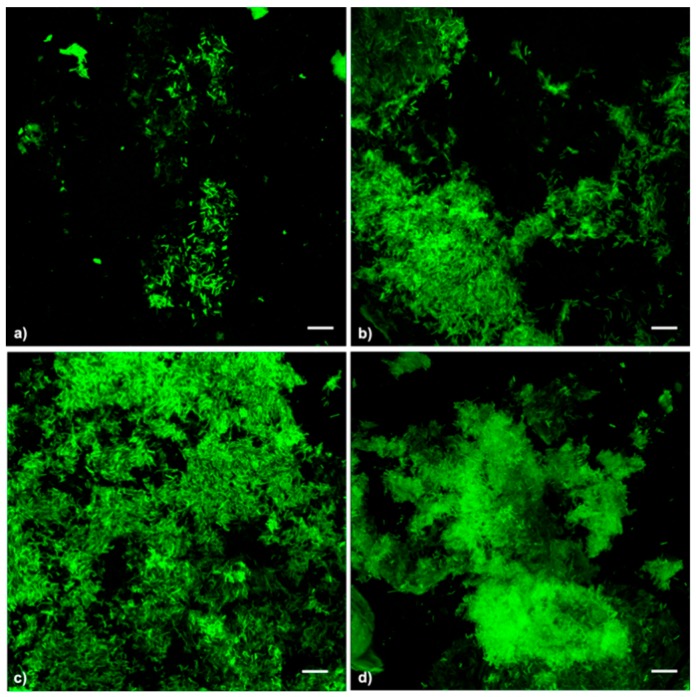
Confocal laser scanning imaging of Xf-DD biofilms under different treatments. (**a**), control; (**b**), pre-treatment; (**c**), post-treatment; (**d**), co-treatment. Scale bar: 10 µm.

Representative biofilm structures observed under different treatments are presented in Figure 4. Biofilm images were obtained by combining z-stack confocal image series with Fiji software. Control samples presented scattered cells and some small bacterial clusters. In all treated Xf-DD biofilms, the biomass increased, forming interconnected clusters with cells oriented horizontally and a few scattered cells were observed. 

### 3.4. NAC Affected the Composition of the Extracellular Polymeric Substances (EPS) 

**Figure 5:**
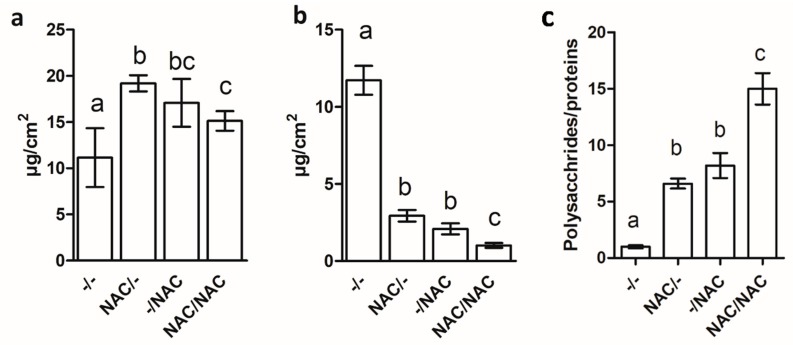
Polysaccharides (**a**) and proteins (**b**) amount in the extracellular polymeric substances (EPS) matrix of Xf-DD biofilm grown for 7 days under different treatments. Panel (**c**) displays the EPS polysaccharides/ EPS proteins ratios. Data represent the mean ± SD of three independent biological replicates. Different superscript letters indicate statistically significant differences (Tukey’s HSD, *p* ≤ 0.05) between conditions. –/–: control, both planktonic and biofilm cells grown without NAC; NAC/–: pre-treatment, only the planktonic cells grown with NAC; –/NAC: post-treatment, only the biofilm cells grown with NAC; NAC/NAC: co-treatment, both planktonic and biofilm cells grown with NAC.

Figure 5a shows the quantification of EPS polysaccharides. The experiments revealed that NAC/– and –/NAC were statistically significantly different compared to the control –/–, with the maximum increase for the treatment NAC/– (72%), followed by –/NAC (53%) and NAC/NAC (26%). 

Figure 5b shows the EPS protein amount. The charts show a statistically significant reduction in the protein amount in all the treatments, with the highest reduction for the treatment NAC/NAC (91%), followed by –/NAC (82%) and NAC/– (75%). 

Figure 5c displays the EPS polysaccharides/EPS proteins ratios. The graph indicated that NAC modulated the chemical composition of the biofilm matrix, with a shift toward the increase of exopolysaccharides versus the extracellular proteins. Indeed, the ratios increased in all the treatments, reaching the maximum increase for the NAC/NAC treatment (+14.7-fold).

### 3.5. NAC Increased the Oxidative Stress within Xf-DD Biofilm

**Figure 6:**
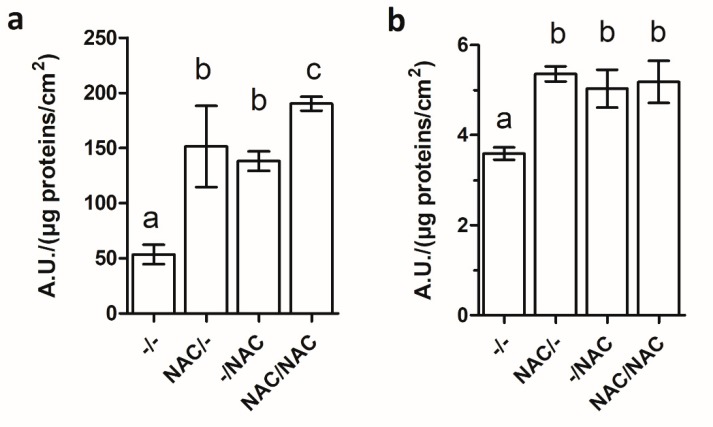
Intracellular (**a**) and extracellular (**b**) level of ROS within Xf-DD biofilm grown for 7 days under different conditions. Data represent the mean ± SD of three independent biological replicates. Different superscript letters indicate statistically significant differences (Tukey’s HSD, *p* ≤ 0.05) between different conditions. –/–: control, both planktonic and biofilm cells grown without NAC; NAC/–: pre-treatment, only the planktonic cells grown with NAC; –/NAC: post-treatment, only the biofilm cells grown with NAC; NAC/NAC: co-treatment, both planktonic and biofilm cells grown with NAC.

Figure 6 shows that Xf-DD biofilms exposed to 500 µM NAC were more prone to accumulate ROS, both intracellularly and extracellularly (in the EPS matrix). Intracellular levels of ROS (Figure 6a) showed the highest statistically significant increase compared to the control –/– in the treatment NAC/NAC (3.5-fold), followed by NAC/– (2.8-fold) and –/NAC (2.6-fold).

Inside the EPS (Figure 6b), a statistically significant increase of ROS level compared with the control –/– was observed in all treatments, with the highest value reported for NAC/– (1.5-fold), followed by NAC/NAC (1.4-fold) and –/NAC (1.4-fold). No statistically significant differences resulted among the treatments. 

### 3.6. NAC Decreased Xf-DD Biofilm Detachment

**Figure 7:**
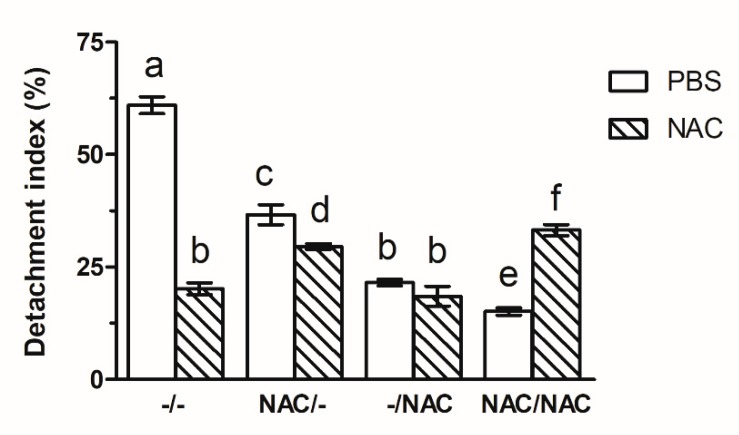
Detachment index of Xf-DD biofilm grown under different conditions after 24 h PBS or NAC treatment. Data represent the mean ± SD of three independent biological replicates. Different superscript letters indicate statistically significant differences (Tukey’s HSD, *p* ≤ 0.05) between different conditions. –/–: control, both planktonic and biofilm cells grown without NAC; NAC/–: pre-treatment, only the planktonic cells grown with NAC; –/NAC: post-treatment, only the biofilm cells grown with NAC; NAC/NAC: co-treatment, both planktonic and biofilm cells grown with NAC.

In order to study Xf-DD biofilm detachment after NAC treatments, an experiment was carried out in which mature biofilms, grown without and with NAC according to the pre-, post- and co- treatments, were soaked in both PBS and 500 µM NAC. After 24 h the biomass remaining on the membrane surface and those released in the bulk liquid were quantified. The tendency towards biofilm detachment for the control Xf-DD biofilm soaked in PBS was 3-fold higher than that soaked in NAC, suggesting that NAC increased Xf-DD cell-cell adhesion (Figure 7). Comparing to the control samples soaked in PBS, none of the pre-, post- and co-treatments promoted biofilm detachment. 

### 3.7. NAC Affected the Level of ROS after Biofilm Detachment 

**Figure 8:**
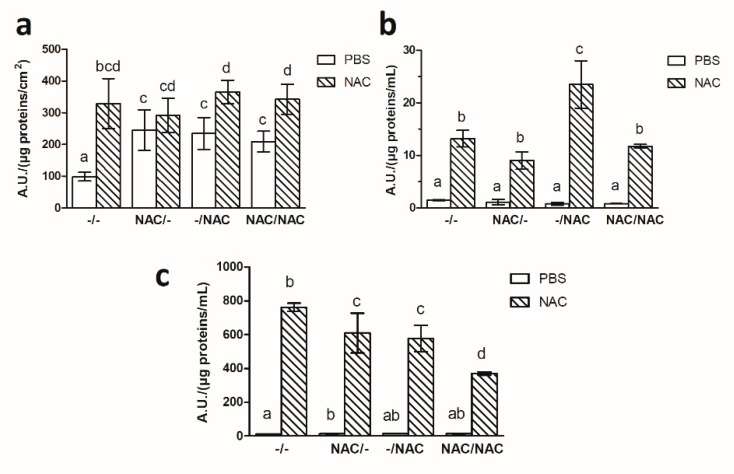
ROS levels within the biofilm remained on the membrane (**a**), detached from the membrane (**b**) and that in the bulk liquid (**c**) after 24 h in presence of phosphate buffered saline solution (PBS) or NAC. Data represent the mean ± SD of three independent biological replicates. Different superscript letters indicate statistically significant differences (Tukey’s HSD, *p* ≤ 0.05) between different conditions –/–: control, both planktonic and biofilm cells grown without NAC; NAC/–: pre-treatment, only the planktonic cells grown with NAC; –/NAC: post-treatment, only the biofilm cells grown with NAC; NAC/NAC: co-treatment, both planktonic and biofilm cells grown with NAC.

After detachment experiments, the level of ROS was quantified in Xf-DD biomass remained on the membrane, in the biomass detached from the membrane and in the bulk liquid medium. The data revealed that the biomass retained on the membrane (Figure 8a) after soaking in 500 µM NAC was more prone to accumulate ROS in comparison to the corresponding ones soaked in PBS, with the maximum increase in the control treatment –/– (3.3-fold), followed by –/NAC (1.6-fold), NAC/NAC (1.6-fold) and NAC/– (1.2-fold). The bulk biomass displayed the same trend (Figure 8b). Indeed, the highest result was obtained for the treatment –/NAC (30.1-fold), followed by NAC/NAC (14.5-fold), –/– (9.0-fold) and NAC/– (8.0-fold). ROS levels in the bulk liquid showed the highest statistically significant increase compared to the negative controls for the treatment –/– (67.2-fold), followed by NAC/– (44.5-fold), –/NAC (43.1-fold) and NAC/NAC (28.8-fold) (Figure 8c).

## 4. Discussion

Many in-vitro studies have demonstrated NAC antibiofilm activity against a variety of Gram-positive, such as *Enterococcus faecalis* and *Staphylococcus aureus,* and Gram-negative pathogenic bacteria such as *Pseudomonas aeruginosa*, *Stenotrophomonas maltophilia* and *Burkholderia cepacia*, as well as some yeasts [26,42,43,44]. Apart from its antibacterial effect, NAC at lethal concentrations reduces bacterial adhesion, inhibits extracellular polysaccharides production and promotes biofilm dispersion in skin pathogens and bacteria associated with chronic diseases in humans [45,46,47]. The encouraging in-vitro results obtained with NAC, the low cost, and the low toxicity to humans and the environment prompted Muranaka et al. [29] to test NAC against *X. fastidiosa* strain 9a5c isolated from symptomatic sweet orange trees. Indeed, both in-vitro and in-vivo findings indicated that NAC at lethal concentrations can be an effective strategy for attenuating the CVC symptoms in citrus. However, downstream from the treated area, there is likely to be a gradient of the biocide concentration, ranging from the prescribed concentration to virtually zero. The response of the plant pathogens to this low, non-lethal concentration of NAC was the impetus of this study. 

For the first time, this work investigates the non-lethal effects of 500 µM NAC on Xf-DD biofilm by simulating pre- (NAC/–), post- (–/NAC) and co- (NAC/NAC) treatments in-vitro. The 500 µM NAC concentration was selected after screening the response of Xf-DD to a range of non-lethal NAC concentrations, where an NAC concentration of 500 µM reduced Xf-DD surface adhesion by 47% in microtiter assays. Interestingly, the best anti-adhesion performance of NAC was obtained at the specific threshold level of 500 μM, which did not correspond to the maximum concentration tested. The non-linear response patterns depicted by the anti-adhesion assays followed a parabola-like shape profile resembling a hormetic property, a situation in which the response to a compound varies with the exposure level. A biphasic profile is common in the biofilm world [48,49]. For instance, antibiotics act in a concentration-dependent manner, where upper and lower threshold concentrations trigger the formation of a biofilm [50].

While microtiter techniques are more suitable for studying bacterial adherence to a surface, the static nature of these techniques has a tendency towards cell nutrient exhaustion that limits the generation of mature biofilm [51]. Therefore, to investigate the effects of the compound on mature biofilms, subsequent experiments on biofilm development and physiology using the transwell system were conducted at 500 µM NAC. A dramatic increase in biofilm biomass was observed after the treatment of 500 µM NAC, with a maximum increase of 78% in the co-treatment (NAC/NAC). BacLight assays demonstrated that the cells within the treated biofilms were viable, confirming the non-lethal activity of NAC. The increase in biofilm biomass under the NAC treatment was also corroborated by CLSM images, where the co-treatment (NAC/NAC) promoted the highest levels of Xf-DD biomass. It is worth noting that a reduced number of adhered cells did not guarantee a biofilm biomass reduction. Glasenapp et al. [36] observed that although sub-lethal concentrations of mangrove extracts reduced microbial adhesion to the surfaces, they stimulated EPS production with a consequent increase in biofilm biomass.

Moreover, biofilms exposed to NAC were more prone to accumulate both intracellular and extracellular ROS. Although NAC is well known for its antioxidant activity, it can act as a pro-oxidant under specific conditions [52]. NAC generates ROS in reactions with transition metals such as Fe^3+^, which is reduced to its catalytically-active form Fe^2+^. Furthermore, NAC can react with free radicals and become a thiyl radical itself [52,53]. In *X. fastidiosa*, ROS acted as an environmental cue that stimulated biofilm development during the early stage of plant colonization, such that the oxidative imbalance was actually required by *X. fastidiosa* to achieve maximum xylem colonization [54]. It has also been proved that *X. fastidiosa* biofilm is related to the sensing of a redox imbalance via the oxidative stress regulator OxyR [54,55]. In fact, *oxyR* mutants were impaired in their ability to attach to surfaces, to aggregate with other cells, and to consequently form biofilm. Thus, biofilm formation by *X. fastidiosa* might be an adaptive response to the exogenous oxidative stress normally encountered by the bacterium in the xylem vessels [54,55,56,57]. In line with the previous considerations, it seems likely that the non-lethal concentration of 500 µM NAC increases the levels of ROS, providing the selective pressure for hyper-biofilm-forming phenotypes. Indeed, the increase in biofilm biomass upon exposure to NAC might have been an adaptive mechanism adopted by the bacteria to protect themselves from the deleterious effect of ROS. According to previous studies, several bacteria respond to sub-lethal doses of ROS by increasing biofilm formation [39,58,59,60]. The NAC promotion of biofilm formation was previously described by Yin et al. [61], where they showed an increasing biofilm formation in *Staphylococcus aureus*, *Enterococcus faecalis*, and *Pseudomonas aeruginosa* caused by the combination of NAC and serum transferrin. The authors demonstrated that biofilm potentiation is caused by a disturbance of the redox status in the bacterial cells. Eroshenko et al. [47] also found that *S. aureus* and *E. faecalis* biofilms increased when exposed to sub-lethal concentrations of NAC.

All NAC-treated samples showed an increase in the EPS polysaccharides/EPS proteins ratios. Indeed, the ratio was shown to increase with the protraction of NAC exposure, with ratios from 1:1 in the control biofilm to 15:1 in the case of NAC/NAC. This result suggests that increased exposure to sub-lethal NAC levels promoted polysaccharide production in the EPS matrix. Further, polysaccharides increase the cohesiveness of the biofilm thereby reducing detachment [62], as seen in our work. Accordingly, Olofsson et al. [63] reported a change in the polysaccharide composition of *Klebsiella* pneumoniae biofilm after being exposed to NAC. In addition, Yin et al. [61] showed an increase in the transcripts of the genes that control the production of extracellular polysaccharides in NAC-exposed *S. aureus*. The polysaccharide overproduction in the EPS matrix after NAC exposure could be related to the oxidative imbalance in the treated biofilm. It has been demonstrated that EPS polysaccharides played a critical role in relief of the oxygen stress in several microorganisms such as *Pseudomonas putida*, *Sinorhizobium meliloti* and *Acinetobacter oleivorans* [59,64,65,66]. Indeed, some polysaccharides can scavenge hydroxyl radicals, inhibiting lipid and protein oxidation [67,68]. Interestingly, the role of the redox-sensing transcription factor OxyR in the regulation of EPS exopolysaccharide production has been reported [65,69]. Burbank and Roper [69] demonstrated that Δ*oxyR* mutants negatively regulated the gene expression of exopolysaccharides, making the mutants more prone to oxidative stress. Besides providing protection against ROS and other environmental injuries, the polysaccharide component allows the cells to adhere to each other and to the surfaces [70]. In *X. fastidiosa*, exopolysaccharide production is fundamental to surface attachment, biofilm formation within the xylem vessels, virulence and insect transmission [71,72]. By knocking out two genes implicated in the exopolysaccharide biosynthesis in *X. fastidiosa* subsp. *fastidiosa*, Killiny et al. [73] demonstrated that mutants were severely constrained in forming biofilm, in moving through the xylem and in being transmitted by the insect, resulting in avirulent phenotypes. In contrast to polysaccharides, EPS protein significantly decreased by up to 91% in all treated biofilm, especially in the co-treatments (NAC/NAC). The disulphide breaking activity of NAC might have led to the destabilization of proteins in the EPS matrix [74]. 

The impact of NAC treatment on Xf-DD biofilm detachment was also examined. The results indicated that none of the NAC-based treatments increased biofilm detachment, compared to the control samples. Rather, control samples (–/–) that were further exposed to NAC became less prone to biofilm disaggregation and detachment. 

Overall, our findings indicated that 500 µM NAC had generated phenotypic diversification in Xf-DD biofilm, promoting biofilm formation (hyper-biofilm-forming phenotype) and discouraging biofilm detachment (hyper-attachment phenotype), while increasing oxidative stress level in the biofilm. This results are in line with a number of studies reporting how exposure to sub-lethal doses of antibiotics may enhance biofilm formation in a wide range of species [75,76]. Despite the undesirable effect of biofilm biomass increase, the hyper-attachment phenotype might be associated with an Xf-DD inability to migrate in xylem vessels to cause a disease. It has been proposed that *X. fastidiosa* forms a biofilm to partially attenuate its own virulence by limiting its diffusion within the plant [11]. Newman et al. [77] observed a reduced virulence in *X. fastidiosa* biofilm with a high degree of adhesion, which inhibited the acquisition and the transmission of pathogens by sharpshooter vectors as well as the fast colonization of the plant throughout the xylem vessels. Thus, this study provides some important implications on the ecology of Xf-DD biofilm, as well as how this plant pathogen should be treated. 

Future works will be devoted to deciphering the mechanisms behind the non-lethal effects of NAC on Xf-DD. Further in-planta investigations are necessary to elucidate the effects of non-lethal concentrations of NAC on Xf-DD virulence.
